# Genotype-by-environment interactions influence the composition of the *Drosophila* seminal proteome

**DOI:** 10.1098/rspb.2023.1313

**Published:** 2023-09-13

**Authors:** Valérian Zeender, Sibylle Pfammatter, Bernd Roschitzki, Steve Dorus, Stefan Lüpold

**Affiliations:** ^1^ Department of Evolutionary Biology and Environmental Studies, University of Zurich, 8057 Zurich, Switzerland; ^2^ Functional Genomics Center Zurich, University of Zurich/ETH Zurich, Winterthurerstrasse 190, 8057 Zurich, Switzerland; ^3^ Center for Reproductive Evolution, Department of Biology, Syracuse University, Syracuse, NY 13244, USA

**Keywords:** genotype × environment, isolines, nutrition, ejaculate protein composition, mass spectrometry-based proteomics, Tandem Mass Tag quantification

## Abstract

Ejaculate proteins are key mediators of post-mating sexual selection and sexual conflict, as they can influence both male fertilization success and female reproductive physiology. However, the extent and sources of genetic variation and condition dependence of the ejaculate proteome are largely unknown. Such knowledge could reveal the targets and mechanisms of post-mating selection and inform about the relative costs and allocation of different ejaculate components, each with its own potential fitness consequences. Here, we used liquid chromatography coupled with tandem mass spectrometry to characterize the whole-ejaculate protein composition across 12 isogenic lines of *Drosophila melanogaster* that were reared on a high- or low-quality diet. We discovered new proteins in the transferred ejaculate and inferred their origin in the male reproductive system. We further found that the ejaculate composition was mainly determined by genotype identity and genotype-specific responses to larval diet, with no clear overall diet effect. Nutrient restriction increased proteolytic protein activity and shifted the balance between reproductive function and RNA metabolism. Our results open new avenues for exploring the intricate role of genotypes and their environment in shaping ejaculate composition, or for studying the functional dynamics and evolutionary potential of the ejaculate in its multivariate complexity.

## 1. Introduction

In many species, the functioning and fertilizing capacity of sperm is heavily influenced by the seminal fluid, a complex mixture of proteins, lipids, immune and glandular cells, hormones, glycoproteins, sugars and water [1]. Particularly the seminal fluid and sperm proteins (hereafter SFPs and SpPs, respectively) have been shown to affect the motility, storage, capacitation and fertilizing ability of sperm, mating plug formation, or the immune and physiological response of the female after mating [2,3].

Ejaculate proteins vary markedly between species and are believed to evolve rapidly via post-mating sexual selection and sexual conflict [4–7]. Yet, an unresolved question is what maintains variation within populations, particularly considering that persistent directional selection on male fitness traits, as often predicted under sexual selection, should in principle erode genetic variation (*sensu* lek paradox: [8]). Apart from potential epistatic and pleiotropic effects [7,9], an important contributor to variation might be the mere complexity and multivariate nature of ejaculates, which provide many different fitness optima. Variation can further be maintained by the complex, often non-transitive fitness outcomes resulting from intricate phenotypic and molecular interactions between ejaculate traits of competing males (e.g. sperm traits or SFPs) and female characteristics (e.g. reproductive tract morphology and physiology, or SFP receptors) [10–12]. However, a prerequisite for understanding these interactions or the fitness landscape of ejaculate proteins is baseline estimates of the standing genetic variation that governs whole-ejaculate functional variation (i.e. beyond selected candidate proteins) [13].

Variation in the ejaculate composition can also arise through plastic responses to varying environmental conditions [2,14]. Much of this plasticity may be related to costs of producing sperm and seminal fluid [15–18], age [19,20] and the fact that not all males can sustain equal costs (e.g. [21]). The resulting ejaculate composition and associated fertilization prospects may thus vary with the phenotypic condition of males [8], and it could further affect offspring development and fitness via the so-called paternal effects [22]. Paternal effects can occur when molecules from the male are either incorporated into eggs or affect female resource allocation in a condition-dependent way [23].

Varying responses of genotypes across environmental conditions (i.e. genotype × environment, G × E, interactions [24]) are thought to form a principal source of standing genetic variation in traits under intense directional selection [25], probably including ejaculate traits [26]. Yet, only a few studies have examined the joint effects of both environmental and genotypic variation on reproductive proteins [27,28], and we are unaware of any study to do so for a broad array of reproductive proteins, let alone the whole-ejaculate proteome.

Here, using *Drosophila melanogaster* as a model system, we quantified the relative amounts of SpPs and SFPs that males of different genotypes and phenotypic condition transfer to standard females. To this end, we manipulated the resources available to developing males, allowed these males to mate with a virgin female after reaching sexual maturity, and then identified ejaculate proteins from the female bursa copulatrix immediately after copulation. Although arthropod ejaculates can also be affected by adult diet [29], we manipulated larval diet as the metabolism, tissue growth and reproductive function of holometabolous insects are particularly sensitive to diet during development [30–35]. Larval diet manipulation may therefore affect a male's resource allocation to sexual traits, including costly ejaculate components at least in early adult life [36–39]. For instance, Macartney *et al.* [33] demonstrated that the same larval diets as used here resulted in poor-diet males transferring an average of 7.60–26.70% fewer sperm than control males at any of four consecutive matings in rapid succession, if they mated that frequently at all. Further, the metabolic costs of biosynthesis can vary between amino acids [40], and ejaculate proteins differ in their function [3]. Males may thus optimize their investments between ejaculate proteins depending on their relative costs and benefits to fitness, contingent on male condition.

Precise predictions of how such condition-dependent modifications should manifest in the whole-ejaculate proteome is challenging owing to missing information on production costs and combinatorial fitness effects of most ejaculate proteins. However, we hypothesized that (i) male genotypes vary in their ejaculate protein composition, (ii) low-diet males generally transfer smaller quantities of particular ejaculate proteins than high-diet males, and (iii) diet-related changes in the ejaculate proteome differ between genotypes, indicating G × E interactions.

## 2. Material and methods

### (a) Study animals

We derived all individuals from 12 independent isogenic lines (hereafter: ‘isolines’) of *D. melanogaster*, created through 15 generations of full-sibling inbreeding from an outbred population of the LHm wild-type strain (approx. 1000 adults with overlapping generations) [41,42]. Previous studies have reported significant genetic variation in ejaculate traits across these isolines [12,33,41]. Using isogenic lines allowed us to subject multiple individuals of each genotype simultaneously to different treatments, thereby separating genotypic from treatment effects. To establish the different developmental treatments, we transferred groups of 40 first-instar larvae to food vials, filled with either standard cornmeal medium (75 g glucose, 100 g fresh yeast, 55 g cornmeal, 8 g agar, 10 g flour, 15 ml Nipagin antimicrobial agent per litre medium) or a nutrient-restricted, less favourable diet. For the latter, we diluted (ninefold) the standard medium in water and agar to a final agar concentration of 15 g l^−1^. The standard females developed on the standard diet at around 200 individuals per culture bottle to minimize possible female size-dependent strategic sperm or SFP allocation [43,44]. We collected virgin females within 8 h of emerging, and focal males once a day, and housed them in vials of 20 individuals, separated by sex, isoline, treatment and day of emergence. We maintained all larvae and flies at 24°C, 60% humidity, and a 14 : 10 light : dark cycle.

### (b) Mating assays

For all experimental matings, all focal males were 4–5 days old, when accessory glands are about fully developed [45]. Each male had mated once on the previous day with a non-experimental virgin female to avoid potential ‘virgin effects’ [46]. We paired each male with a 3-day-old standard female and, within 10 min of terminating copulation, jointly transferred them to a microcentrifuge tube to snap-freeze (in liquid N_2_) and stored them at −80°C for later dissection. We completed at least nine matings per isoline-treatment combination on each of 10 consecutive days.

### (c) Dissections and tissue isolation

For each of the 24 isoline-treatment combinations, we dissected 90 mated females (total *n* = 2160) on ice under a Leica MS5 stereomicroscope (Leica Microsystems, Heerbrugg, Switzerland) at 40 × magnification, in phosphate buffered saline (PBS) supplemented with 2% sodium dodecyl sulfate (SDS) and 5% dithiothreitol (DTT) (weight/volume %). After extracting their lower reproductive tract, we retained only the bursa copulatrix, cleared of the seminal receptacle, spermathecae, parovaria and associated fat body. We did so to minimize contamination by secretions from these female tissues [47]. Since sperm enter the different storage organs after the end of copulation and females do not typically eject excess sperm for several hours in *D. melanogaster* [48,49], the bursa copulatrix immediately after mating provided a representative ejaculate sample. We pooled groups of 10 bursae per combination in microcentrifuge tubes and stored them at −80°C until further processing. Additionally, to determine the diet effect on male phenotype and to rule out possible female size-mediated ejaculate tailoring [43,44], we measured the thorax length of 35 focal males and standard females per isoline-treatment combination to the nearest 0.025 mm using a reticular eyepiece (see electronic supplementary material for details about thorax length analysis).

### (d) Sample preparation and Tandem Mass Tag labelling

We distributed the 90 bursae per combination across three biological replicates, resulting in a total of *n* = 72 samples, each containing 30 bursae, which we analysed across five quantification experiments using tandem mass spectrometry (MS/MS) with 16-plex Tandem Mass Tag (TMT, Thermo Fisher) labelling (electronic supplementary material, figure S1).

For protein extraction, samples were mechanically solubilized in PBS with a pestle (2% SDS, 5% DTT), and proteins were acetone-precipitated and resuspended in iST-NHS buffer (iST-NHS kit, PreOmics). After protein digestion, peptides were labelled with a 1 : 2.5 TMT16 tag ratio (peptide : label) before resuspension in 3% acetonitrile/0.1% formic acid. We equally combined the individual samples and fractionated offline the TMT pools using high-pH reverse-phase chromatography. Each TMT experiment consisted of 20 liquid chromatography–mass spectrometry (LC-MS/MS) runs on a Thermo Scientific Fusion Lumos mass spectrometer equipped with a Waters M-Class LC system. We followed each scan by a data-dependent MS/MS scan and isolated the most abundant fragments. We assigned reporter ion such that they reduced the effect of cross-population interference (i.e. channel leakage; electronic supplementary material, figure S1; [50]) and increased quantitative accuracy of proteins across isobaric labels [51]. Detailed information can be found in the electronic supplementary material.

### (e) Protein identification and quantification

We processed the raw data in Proteome Discoverer v. 2.5 (Thermo Fisher Scientific), searching against the *D. melanogaster* protein database containing only the longest isoform for each protein (r6.32; [52], *n* = 13 813 entries). To increase the reliability of protein identification for quantification, we considered only those proteins for which at least two proteotypic peptides were detected. We assigned spectra using a precursor mass tolerance of 20 ppm and fragment ion tolerance of 0.5 Da using the Sequest algorithm [53]. Static modifications included a specific cysteine modification (acetylhypusine, +113.084 Da), TMT (+304.207 Da) on the peptide N-terminus and lysine residues. Variable modifications included an acetylation on the N-terminus of the protein end (+42.011 Da), oxidized methionine (+15.995 Da) and methionine loss with (−131.040 Da) and without acetyl (−89.030 Da). Enzyme specificity was set to trypsin allowing a minimal peptide length of six amino acids and a maximum of two missed cleavages. The maximum false discovery rate (FDR) for peptides was set to 0.01. For reporter ion quantification, the integration tolerance was 20 ppm for the most confident peak. Protein fold changes were computed based on Intensity values reported in the protein output.

Proteome Discoverer identified *n* = 4328 unique proteins across the five TMT experiments (electronic supplementary material, table S1 and figure S2), which were exported to R v. 4.1.2 [54] for all further analyses. On average across all TMT runs, proteins of male origin constituted 40.44% and those of female origin 51.51% of the detected proteins, while 18.98% were shared between sexes (electronic supplementary material, figure S2). To maximize coverage across TMT experiments, we imputed for any protein with a single missing identification (*n* = 445) the mean diet-specific abundance of the other four TMT experiments. To be conservative, we excluded all proteins with missing identification in more than one TMT experiment (*n* = 1336 proteins). This resulted in a final dataset of *n* = 2992 unique proteins (electronic supplementary material, table S1). Batch effects between TMT experiments were removed using a *ComBat*/SL/TMM/log_2_ normalization procedure (detailed in the electronic supplementary material, figure S3 and table S1). We then selected those proteins that overlapped with the *D. melanogaster* sperm proteome (*n* = 2288; [55]), or with either the high-confidence (*n* = 311) or candidate set of SFPs (*n* = 314) [7]. Note that *n* = 188 of these proteins had shared IDs between SpPs and SFPs. Of the *n* = 1284 proteins that overlapped with the previously published lists of ejaculate proteins, *n* = 636 have also been recorded in the female reproductive tract (FRT) [56], thus leaving their origin unclear. We restricted our dataset to those *n* = 648 (i.e. *n* = 1284 − 636) proteins that, to our knowledge, were uniquely male-derived and transferred during copulation (henceforth referred to as ‘working dataset’). The working dataset was complemented with 24 candidate SpPs and 27 candidate SFPs based on gene expression profiles in FlyAtlas2 [57]. Twenty-two of these SFPs also expanded another recent list of candidate *D. melanogaster* SFPs [58], in addition to [7]. To be conservative, we considered proteins as candidates if their genes were strongly overexpressed (top 10%) in males relative to females and were additionally strongly biased towards either accessory glands or testes among the male tissues (i.e. most extreme 10% on either side; for details on the procedure see the electronic supplementary material, figure S4). Adding these 51 candidate proteins to our working dataset resulted in an ‘extended working dataset’ of *n* = 699 proteins, which consisted of 140 SFPs, 433 sperm proteins, 75 proteins with dual annotation and 51 candidate ejaculate proteins (electronic supplementary material, table S1). All analyses are based on the ‘extended working dataset’.

### (f) Effects of diet and genotype

To investigate differentially expressed proteins for the high−low diet contrast, we fitted a mixed effects model with the normalized abundances as the response variable, diet and isoline as fixed effects and biological replicates as a random effect, using the *build_model* function (*prolfqua* package [59]). To test for diet-dependent enrichment of genes that encode ejaculate proteins, we ranked proteins by their *t*-statistics obtained from the high−low diet contrasts. We then subjected these proteins to a gene set enrichment analysis (GSEA) using the *gseGO* function (*clusterProfiler* package [60]), with the full *D. melanogaster* proteome as a background (*org.Dm.eg.db* package [61]), considering categories as enriched if their Benjamini–Hochberg adjusted *p*-value was less than 0.05.

We assessed relationships between normalized samples in a principal component analysis (PCA) using the *prcomp* function (*stats* package; [54]). Given their corresponding contribution to total variance (see Results), we used only the first two principal components (i.e. PC1 and PC2) as response variables in further analyses. We investigated the variance explained by the experimental factors on PC1 and PC2 using type-III ANOVAs (which are suitable for models with interaction terms) as implemented in the *Anova* function (*car* package [62]), with either PC1 or PC2 as a response variable and diet, isoline and the isoline × diet interaction as fixed effects. We transformed the resulting *F*-values to standardized effect sizes (partial *ε^2^*) with 95% confidence intervals using the *epsilon_squared* function (*effectsize* package; [63]). We used Cohen's [64] benchmarks of *ε^2^* = 0.01, 0.06 and 0.14 to define effects as small, medium and large, respectively. We quantified the contribution of single proteins to either PC1 or PC2 using the *fviz_contrib* function (*factoextra* package [65]). Based on the STRING database (v. 11.5 [66]), we then explored pairwise interactions among the proteins above the expected mean contribution in the working dataset, separately for SpPs and SFPs, retaining only interaction scores greater than 0.9. To further visualize the ANOVA results, we illustrated the relationships between samples in a heatmap using the *pheatmap* function (*pheatmap* package [67]) with the *ward.D* clustering method [68]. Since a previous study [69] estimated the mode of evolution (constrained, positive, relaxed) for the SFPs (but not SpPs), we further mapped these categories onto our heatmaps and tested for links between mode of evolution and differential protein abundances.

## 3. Results

### (a) Variation in body size

Diet and isoline accounted for 63.90% and 9.88% of variation in male thorax length, respectively. Even though males developing on a restricted diet were consistently smaller than their high-diet counterparts (linear mixed-effects model, *n* = 840, *β* = −0.46 ± 0.04 (s.e.), *t* = −11.63, *p* < 0.001; electronic supplementary material, figure S5), the relative abundances of different protein types (SpP, SFP, FRT or combined) differed by ≤0.5% between diet treatments (electronic supplementary material, figure S6). Between treatments, the difference in thorax length of the randomly assigned standard females (*F*_1,816_ = 2.83, *p* = 0.093, *ε*^2^ = 0.03 [95% confidence limits: 0.01, 1.00], electronic supplementary material, figure S5) was negligible compared with that of the experimental males (*F*_1,816_ = 230.70, *p* < 0.001, *ε*^2^ = 0.71 [0.69, 1.00], electronic supplementary material, figure S5).

### (b) Extensive ejaculate coverage and novel empirical evidence for transfer of candidate seminal fluid proteins

We characterized the protein profiles of whole ejaculates as transferred by males during mating across 12 isolines, each reared on two larval diets. Each of the five TMT experiments revealed 3185−3608 proteins (see electronic supplementary material for details on the ejaculate coverage). The proteins identified in the female bursae included *n* = 140 of those that had previously been classified as putative SFPs [7,55], which we here confirmed to indeed be transferred during copulation. This estimate is still conservative because it excludes an additional 160 candidate SFPs that were detected in the bursa but shared annotation with the FRT proteome, thus leaving their origin ambiguous. Furthermore, out of the 51 newly identified candidate ejaculate proteins, 13 have been reviewed in the UniProt Knowledgebase and primarily classified as enzymes or humoral factors, and another two have been annotated as seminal fluid proteins of unknown function (E1JHF6 and M9PEY3, electronic supplementary material, table S1 [70]).

### (c) Genotypic and diet-related variation in ejaculate proteome

**Figure 1.**
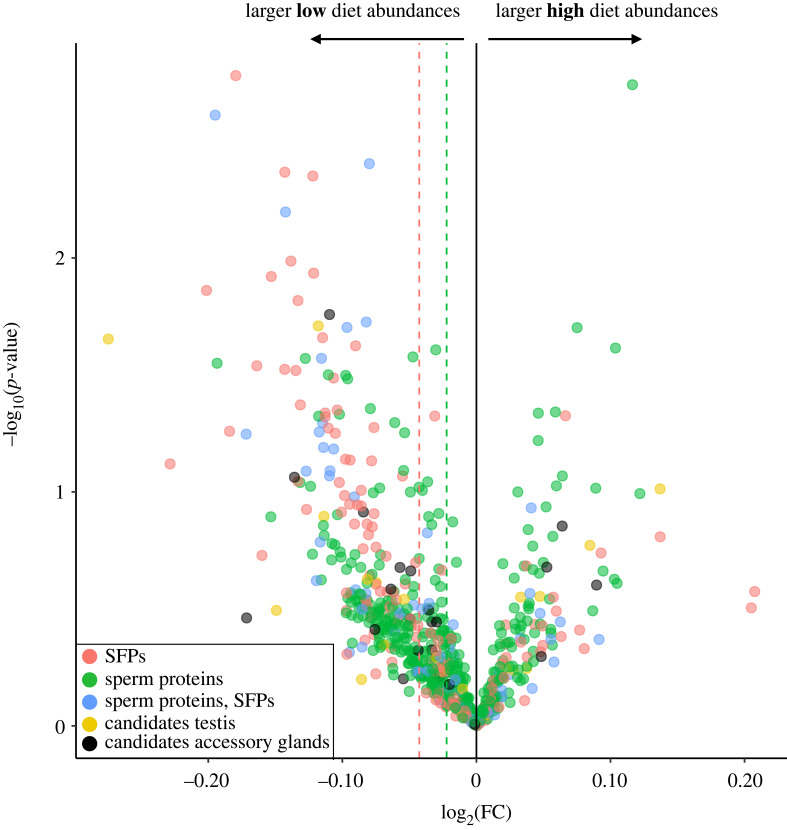
Volcano plot showing the changes in abundance in the ejaculate protein composition for the high–low diet contrast. No protein differed significantly in abundance after correction for multiple testing. SFPs are coloured in red, sperm proteins in green, and proteins with dual annotation in blue. The mean directional fold change for sperm proteins and SFPs is indicated by the green and red dotted line, respectively.

There was no significant change in the abundance of any protein in response to diet across isolines after FDR correction (figure 1). Although not passing the FDR-corrected significance threshold, most proteins uniquely annotated as either SFP or SpP were biased toward low-diet males (SFPs: 105 versus 35; SpPs: 308 versus 125), with no proportional difference between protein types (*χ*^2^_1_ = 3.07, *p* = 0.38). The mean directional change was a log-fold change of −1.03 and −1.02 for SFPs and SpPs, respectively (figure 1).

**Figure 2.**
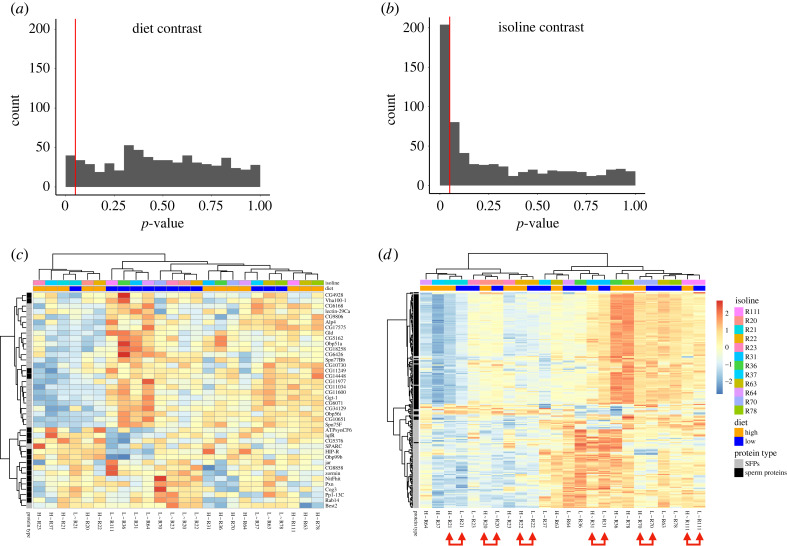
Clustering of the ejaculate protein filtered for significant contrasts for diet and isoline. (*a*,*b*) Distribution of the ANOVA *p*-values filtering for the diet contrast (*a*) or isoline contrast (*b*). The red line in both histograms indicates the significance threshold (*p* = 0.05), with 40 and 203 proteins, respectively, below this cut-off before FDR correction. (*c*,*d*) Heatmaps of significant ANOVA results from (*a*) and (*b*), filtering for the diet contrast (*c*) or isoline contrast (*d*). The hierarchical clustering of isoline and diet (columns), and of ejaculate protein (rows), is based on *ward.D2* clustering [68]. Heat map colours indicate the relative change in abundance for each protein (rows). The red arrows annotate the six isolines having their ejaculate profiles clustering together between the two diets. Labels at the bottom indicate diet treatments (L, low diet; H, high diet) and isoline ID's (e.g. ‘R70’). The labels of proteins in (*c*) and (*d*)—omitted here for spatial reasons—are available in electronic supplementary material, table S2.

Next, still on the extended working dataset, we compared the diet and isoline contrasts. Although none of the *p*-values were statistically significant after FDR correction, we explored more closely those proteins that were differentially expressed relative to diet or isoline contrasts prior to FDR correction (*n* = 40 and 203, respectively; figure 2*a,b*). Focusing on the diet contrast only, proteins formed no clear low- or high-diet clusters (figure 2*c*). When isolating the isoline contrast, six of the twelve isolines showed signs of condition dependence, in that their ejaculate profiles clustered separately (i.e. were different). The remaining six isolines showed high similarity between treatments (figure 2*d*). Additionally, 26 and 188 proteins were present only in the diet or the isoline contrast, respectively, whereas 15 proteins were shared between both contrasts (electronic supplementary material, figure S7*c* and table S2). Proteins further separated by type, with only six SFPs clustering among SpPs and 12 SpPs among SFPs (figure 2*d*). Lastly, the annotation relative to the mode of evolution derived from Patlar *et al.* [69] indicated that most proteins that were differentially abundant between isolines; 55.29% and 34.29% were under relaxed evolution or selectively constrained evolution, respectively (for the diet contrast: 47.62% and 33.33%; electronic supplementary material, figure S7*a,b*).

**Figure 3.**
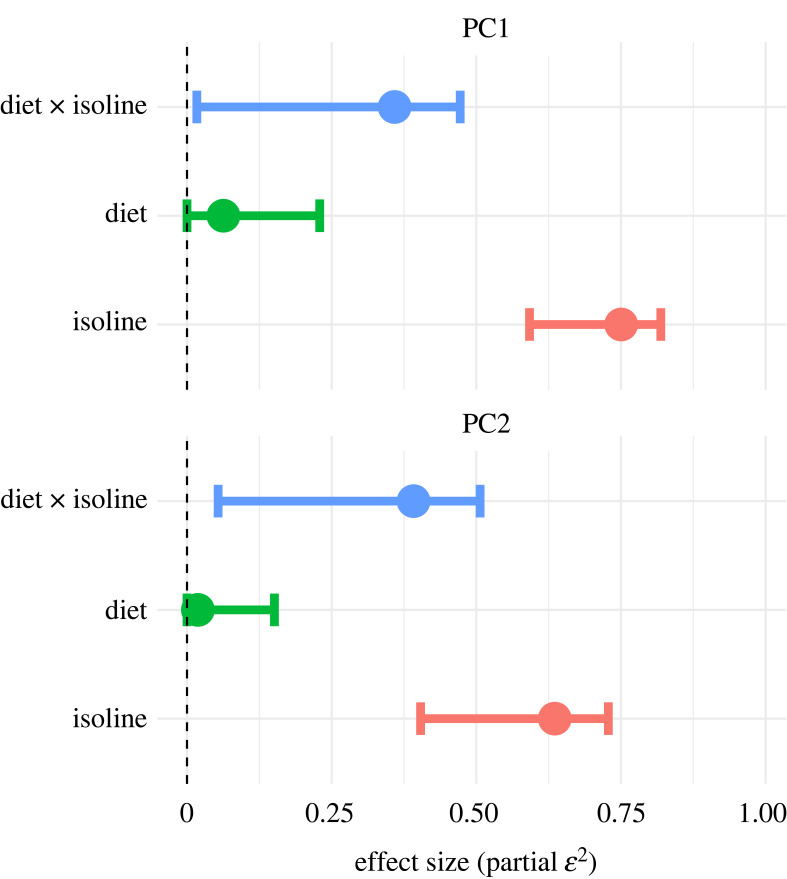
Effect size (partial *ε*^2^ with 95% confidence interval) of the contribution of diet, isoline, and the isoline × diet interaction to PC1 and PC2, respectively. Effect sizes were computed from the *F*-values obtained in type-III ANOVAs, with either PC1 or PC2 as a response variable and the three predictors as fixed effects (see text for details).

A PCA of the transferred ejaculate composition on the working dataset revealed that 39.05% of the variation among samples was explained by the first two PCs, with PC1 capturing 30.37% and PC2 another 8.69% of the total variation (electronic supplementary material, figure S8). The isoline × diet interaction explained a significant portion of variation in both PC1 (ANOVA: *F*_11,48_ = 4.00, *p* < 0.001, *ε*^2^ = 0.36 [0.02, 0.48]) and PC2 (*F*_11,48_ = 4.45, *p* < 0.001, *ε*^2^ = 0.39 [0.05, 0.51]) (figure 3). The main effect of diet was weak and essentially undetectable, with the 95% effect size confidence interval including zero for both PC1 (*F*_1, 48_ = 4.30, *p* = 0.04, *ε*^2^ = 0.06 [0.00, 0.23]) and PC2 (*F*_1,48_ = 1.94, *p* = 0.17, *ε*^2^ = 0.02 [0.00, 0.15]). Isoline explained a substantial part of both PC1 (*F*_11 48_ = 17.12, *p* < 0.001, *ε*^2^ = 0.75 [0.59, 0.82] and PC2 (*F*_11,48_ = 10.36, *p* < 0.001, *ε*^2^ = 0.64 [0.40, 0.73]) (figure 3).

### (d) Gene set enrichment analysis and STRING analysis

**Figure 4.**
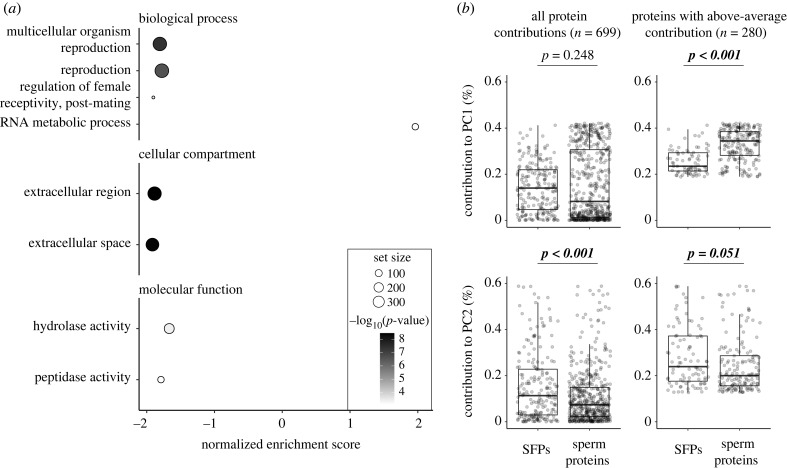
GSEA and protein types to PC1 and PC2. (*a*) GSEA of the high-low diet contrast for all three gene ontologies. (*b*) Contribution of single proteins to PC1 and PC2, split by protein type. Left: taking all proteins into account, SFPs and sperm proteins did not vary in their contribution to PC1, whereas more SFPs contributed to PC2. Right: proteins above the expected average contribution to PC1 are significantly enriched in sperm proteins, whereas in PC2 there was an enrichment in SFPs. The total cumulated percentage of variation explained by the proteins with above-average contribution to PC1 and PC2 is 88.66% and 97.28%, respectively.

The GSEA based on the fold-change ranking for the high- versus low-diet contrast revealed that ejaculates of high-diet males were significantly enriched for gene ontology (GO) annotation associated with RNA metabolic processes, whereas ejaculates of low-diet males were enriched for GO annotation related to reproduction and extracellular enzymatic activity (figure 4*a*; electronic supplementary material, table S2). When examining all proteins that contributed to PC1, there was no significant enrichment in either sperm or seminal fluid, but proteins above the mean contribution to PC1 (*n* = 280) were enriched for SpPs (*β* = 0.07 ± 0.01, *t*_278_ = 8.45, *p* < 0.001; figure 4*b*; electronic supplementary material, table S2). For PC2, there was an enrichment in SFPs when including all proteins (*β* = −0.06 ± 0.01, *t*_721_ = –4.19, *p* < 0.001; figure 4*b*) or, to a lesser extent, among those with an above-average contribution (*β* = −0.05 ± 0.02, *t*_278_ = −1.96, *p* = 0.051; figure 4*b*, electronic supplementary material, table S2).

A STRING network analysis for all SpPs with an above-average contribution to PC1 showed that these proteins formed a network with *n* = 368 edges observed compared with the expected 242 edges (protein–protein interaction enrichment: *p* < 0.001), and there was a STRING local network cluster densely enriched for mostly uncharacterized proteins (electronic supplementary material, figure S9*a* and table S2). The same analysis for SFPs revealed a network with *n* = 68 edges observed relative to 21 edges expected (protein–protein interaction enrichment: *p* < 0.001), and an enrichment for proteins regulating female receptivity and extracellular space (*p* < 0.001; electronic supplementary material, figure S9*b* and table S2), containing well-known proteins such as ovulin, Acp36DE or Acp76A.

## 4. Discussion

Despite important consequences for the adaptive potential, little is known about how genetic and environmental variation interact to affect the ejaculate proteome. A better characterization of the genotypic variation and condition dependence of ejaculate proteins is critical to improve our understanding of the targets of post-mating sexual selection, the processes of post-mating prezygotic reproductive isolation, and the links between male health or quality and fertility [71]. Contrary to theoretical predictions [36–39], we found no compelling evidence for a main effect of diet quality on ejaculate composition. Rather, variation in the ejaculate proteome was explained by genotypes and a genotype-by-diet interaction. Finally, we discovered new candidate ejaculate proteins and provide further evidence in support of being part of the ejaculate to proteins that had previously been considered as putative SFPs [7,55].

It is now relatively well established that functional interactions between the different ejaculate traits can generate complex fitness landscapes with multiple optima [72], and that ejaculate × female interactions can result in non-transitive fitness outcomes [71]. Recent work has also shown a G × E interaction for a set of SFPs at the level of gene expression [27,28]. Here, we empirically added to the multivariate complexity and dynamic nature of ejaculates by documenting an interactive effect of genotype and larval diet on the whole-ejaculate proteome, including the transferred SFPs and SpPs. All these types of interactions, but particularly G × E interactions as shown here, can contribute critically to the maintenance of genetic variation in populations where intense directional sexual selection would be predicted to deplete such variation [25]. The significant differences in the ejaculate proteome between the few isolines examined, combined with the much stronger effect of isoline than diet, are indeed indicative of considerable genetic variation, similar to other ejaculate traits [73]. Yet, further studies on larger sets of genotypes and using quantitative genetic breeding designs are now needed for robust estimates of the population-level variance, narrow-sense heritability or evolvability of the ejaculate proteome.

The sperm proteins loading on PC1, which itself was primarily explained by genotypic variation, consisted of mostly uncharacterized proteins that warrant further investigation. By contrast, the corresponding SFPs included some extensively studied ejaculate proteins such as ovulin, Acp36DE or Acp76A. These proteins are known to affect ovulation, sperm storage and mating plug formation, respectively [74,–76], thereby having well-established fitness consequences. That these proteins were central to one of the main clusters in the protein network could be related to their combined functional significance, but it is also possible that they simply have more experimentally confirmed molecular partners in the literature by being more thoroughly characterized. Note that sex peptide, another well-studied and multi-functional seminal molecule [77–79], was not quantified in our study. With only 36 amino acids in its mature form as transferred to the female [80], this peptide was detected by only a single proteotypic peptide in each TMT run, thus falling below our threshold of two such peptides for reliable identification.

Among those proteins that significantly differed between isolines, sperm proteins, on average, appeared to show more concerted diet-related changes within isolines than did SFPs. The higher synchronization of sperm-bound proteins could be explained by their changing as entire clusters with any sperm added or removed, suggesting that the genetic variation governing differential sperm and SFP abundance is largely distinct. By contrast, SFPs are derived from different secretory organs, mixed in these organs, and added to the seminal fluid during ejaculation, such that individual SFPs may vary somewhat more freely than SpPs. Patlar *et al.* [69] examined the mode of evolution of 317 SFP genes in *Drosophila*. Over half (175/317, 55%) of these proteins were present in our working dataset, and those differing significantly between isolines were predominantly classified as being under-relaxed or selectively constrained evolution [69]. Although these results primarily relate to the sequence of genes or proteins, it seems plausible that the expression might also be relatively constrained for most proteins. Particularly the multivariate and interactive nature of ejaculate proteins, but also their potential co-expression in the same tissue or functional co-dependence, might limit rather than promote the evolution of exaggerated expression of individual components [12]. That ejaculate proteins may often co-vary in their abundance is also supported by reports of genetic correlations in the expression of critical SFP genes in other taxa [27].

While differing to some extent within isolines, there was little evidence that the ejaculate proteome varied in a condition-dependent manner. Even a 90% reduction in nutrients available to developing males did not systematically alter the composition of their ejaculate proteome despite the expectedly high costs of producing ejaculates [15–18]. Yet, while rejecting our prediction, the lack of clear condition dependence is not necessarily surprising. For example, juvenile diet in *D. melanogaster*, often mediated by the resulting body size, has been shown to affect the expression of some [32,33,81] but not of other costly ejaculate traits (e.g. sperm length [82]). Similarly, a recent meta-analysis [29] highlighted the unequal sensitivity of different ejaculate traits to dietary manipulation. Even though seminal fluid quantity (i.e. non-sperm ejaculate size or accessory gland size) appears to be one of the most sensitive ejaculate traits across taxa [29], here we quantified protein composition rather than overall volume. What remains unknown is whether diet effects on ejaculate composition could be more apparent in protein production, after multiple copulations instead of a single mating, or if adult males instead of larvae are manipulated.

If the ejaculate protein composition indeed is not condition-dependent, it may be indicative of genetic canalization [83], buffering the ejaculate against adverse conditions to protect its important reproductive function [84]. If so, our dietary conditions may not have been adverse enough to elicit a reproductive cost in these flies, where, as suggested by electronic supplementary material, figure S6, males from each diet were able to transfer essentially equal amounts of ejaculate (relative to female proteins as a reference). Therefore, it is possible that the seminal fluid differed less between our diet treatments in its composition than in its overall quantity as suggested elsewhere (e.g. [81]), which was masked by our physical and computational sample normalization. Nonetheless, while compositional differences were observed, it remains unclear if standard females exhibit differential FRT allocation based on mate quality. As a result, conclusions about male ejaculate size based on these data remain speculative.

A recent study found no effect of the same diet treatments as here on males' sperm replenishment after depletion, sperm defence ability, ability to delay female remating or their offspring production [33]. Given that the last three of these traits are known to be influenced by ejaculate proteins (e.g. [3]), our results are complementary by correspondingly showing essentially undetectable to no condition dependence in the ejaculate protein composition. Nonetheless, the ejaculate proteome of high-diet males was enriched for RNA metabolic processes and that of low-diet males for enzymatic activity in the extracellular space. These changes could point toward at least some functional shift in the ejaculate proteome relative to male condition even if clear condition dependence in protein abundances was not detected at the treatment level.

Even though our results indicated no clearly detectable condition dependence in the ejaculate protein composition under different dietary regimes, differential ejaculate investment has previously been shown relative to social environment [85,86], female mating status [43,87] and male age [88]. The ejaculate composition may also vary across successive matings, due to either unequal depletion of different ejaculate proteins or strategic ejaculate modulation relative to the depletion status [33]. Indeed, the same diet treatments as ours have previously been shown to reduce sperm transfer of low- compared with high-condition males, but primarily from the second of a series of copulations [33]. The much smaller low-diet males may pay a relatively higher price per unit ejaculate, but the consequences may emerge only when males start depleting ejaculate components. Such differential transfer dynamics seem plausible to the extent that seminal fluid can deplete faster than sperm [18,89]. Further, replenishing the reserves of sex peptide (and possibly of other proteins) can take several days after multiple matings [90], which itself could be influenced by male condition [79].

The direct fitness consequences of variation in the ejaculate protein composition remain to be explored, particularly considering the potential buffering effect of functional redundancies. Many individual SFPs are known to elicit differential reproductive output [91], but tracking the fitness consequences of condition-dependent abundances in single SFPs does not necessarily exclude the possibility that non-focal proteins may also be affected and contribute to eliciting the phenotypic response. Indeed, in a promiscuous species like *D. melanogaster*, the reproductive outcome may depend on the interactions between many actors [12], and the effect of individual, differentially abundant ejaculate proteins may be limited depending on their position in the regulatory or functional network. In fact, a recent review on the effect of SFPs on sperm competitive fitness highlighted the need to couple gene perturbation assays (e.g. knockouts) with fitness assays to unambiguously link reproductive proteins to fitness effects [92]. For example, Patlar & Civetta [93] investigated how particular SFPs affect male reproductive success by knocking out 16 genes encoding well-known SFPs and then subjecting males to competitive mating assays to estimate their sperm competitiveness and female refractoriness. While that study showed that almost all SFPs investigated were important for sperm defence (i.e. P_1_)*,* none of them affected sperm offence (i.e. P_2_) or female refractoriness [93].

That we identified several novel candidate ejaculate proteins relative to recent proteomic studies [55,94] might have resulted from greater sensitivity of our methodology or broader sampling in terms of both independent samples and quantification experiments. For example, each experiment missed a small proportion of proteins, but many of these proteins were recovered by the remaining four experiments, thus supporting their inclusion in the dataset. Furthermore, we extracted the whole-ejaculate proteome directly from the bursa copulatrix, which provided strong evidence of transfer during copulation for any male-derived protein. An important limitation of this approach, however, was that approximately half of the putative ejaculate proteins had previously also been annotated as female-derived [56]. By conservatively excluding these proteins owing to their ambiguous origin, we might have overlooked candidates with condition-dependent male allocation. Isotopic labelling could overcome this issue [95]. Combined with the general challenges of identifying and annotating seminal proteins by origin [58,96], our analyses show that, despite much progress in recent years, much remains to be learnt about the composition, variation, function and evolution of the *Drosophila* ejaculate proteome.

In conclusion, we report at best limited condition-dependent and significant genotypic variation in the ejaculate protein composition. Despite the necessarily challenging logistics, we encourage future studies of the whole-ejaculate proteome in a quantitative genetic framework to better understand its evolvability. We further hope to inspire studies that explore the effects of ejaculate depletion on the seminal protein composition. This will help clarify how different ejaculate components change relative to one another across successive matings, and how this relates to both male condition and reproductive success. Using whole-ejaculate proteomic approaches as opposed to single-protein analyses will also allow us to start understanding the functional dynamics and evolutionary potential of the ejaculate in its multivariate complexity, not as a set of functionally independent traits.

## Data Availability

The mass spectrometry proteomics data were handled using the local laboratory information management system [97], and have been deposited to the ProteomeXchange Consortium via the PRIDE partner repository [98] with the dataset identifier PXD032799. Data and code are available on Dryad Digital Repository at https://doi.org/10.5061/dryad.djh9w0w5m [99]. The electronic supplementary material is accessible via [100].
